# Association between Promoter Methylation of Gene *ERCC3* and Benzene Hematotoxicity

**DOI:** 10.3390/ijerph14080921

**Published:** 2017-08-16

**Authors:** Min Zheng, Feiliang Lin, Fenxia Hou, Guilan Li, Caiying Zhu, Peiyu Xu, Caihong Xing, Qianfei Wang

**Affiliations:** 1 Key Laboratory of Chemical Safety and Health, National Institute for Occupational Health and Poison Control, Chinese Center for Disease Control and Prevention, Beijing 100050, China; zhengm0916@126.com (M.Z.); lfl992@163.com (F.L.); houfenxia66@163.com (F.H.); gui_lanli@126.com (G.L.); 2 Key Laboratory of Genomic and Precision Medicine, Collaborative Innovation Center of Genetics and Development, Beijing Institute of Genomics, Chinese Academy of Sciences, Beijing 100101, China; 3 Department of Nutrition, Food Safety and Toxicology, West China School of Public Health, Sichuan University, Chengdu 610041, China; xpy9929@163.com; 4 State Key Laboratory of Experimental Hematology, Institute of Hematology and Blood Diseases Hospital and Center for Stem Cell Medicine, Chinese Academy of Medical Sciences and Peking Union Medical College, Tianjin 300020, China; zhucy5@163.com

**Keywords:** benzene, occupational exposure, *ERCC3*, methylation, hematotoxicity

## Abstract

Benzene is a primary industrial chemical and a ubiquitous environmental pollutant. *ERCC3* is a key player in nucleotide excision repair. Recent studies suggested that site-specific methylation is a possible mechanism of the transcriptional dysregulation by blocking transcription factors binding. We previously found that the average promoter methylation level of *ERCC3* was increased in benzene-exposed workers. In order to test whether specific CpG sites of *ERCC3* play an important role in benzene-induced epigenetic changes and whether the specific methylation patterns are associated with benzene hematotoxicity, we analyzed the promoter methylation levels of individual CpG sites, transcription factor binding motif and the correlation between aberrant CpG methylation and hematotoxicity in 76 benzene-exposed workers and 24 unexposed controls in China. Out of all the CpGs analyzed, two CpG units located 43 bp upstream and 99 bp downstream of the transcription start site of *ERCC3* (CpG 2–4 and CpG 17–18, respectively), showed the most pronounced increase in methylation levels in benzene-exposed workers, compared with unexposed controls (Mean ± SD: 5.86 ± 2.77% vs. 4.92 ± 1.53%, *p* = 0.032; 8.45 ± 4.09% vs. 6.79 ± 2.50%, *p* = 0.024, respectively). Using the JASPAR CORE Database, we found that CpG 2–4 and CpG 17–18 were bound by three putative transcription factors (TFAP2A, E2F4 and MZF1). Furthermore, the methylation levels for CpG 2–4 were correlated negatively with the percentage of neutrophils (*β* = −0.676, *p* = 0.005) in benzene-exposed workers. This study demonstrates that CpG-specific DNA methylation in the *ERCC3* promoter region may be involved in benzene-induced epigenetic modification and it may contribute to benzene-induced hematotoxicity.

## 1. Introduction

Benzene is a primary industrial chemical and a ubiquitous environmental pollutant present in cigarette smoke and motor vehicle exhaust. Occupational benzene exposure causes toxicity to the hematopoietic system (hematotoxicity), acute myeloid leukemia (AML) and other hematopoietic disorders [1,2,3]. It is well known that the alteration of DNA methylation in leukemia involves genome-wide hypomethylation and gene-specific promoter hypermethylation, which leads to genomic instability. Recent studies reported that hypermethylation in *p15* and hypomethylation in *MAGE-1* were associated with benzene exposure [4], and down-regulation of *p15* and *p16* expression was correlated with hypermethylation in benzene poisoning patients [5]. Study in vitro also reported that the benzene-induced decrease of *PARP-1* mRNA expression might be modulated by promoter methylation [6], and global DNA hypomethylation induced by benzene metabolite hydroquinone may be another mechanism for the leukemogenicity of benzene [7]. These studies suggested that methylation might have an effect on the development of benzene-induced hematotoxicity and carcinogenicity in a manner complementary to direct mutations of the DNA sequence by benzene. 

*ERCC3* is an ATP dependent DNA helicase that is involved in nucleotide excision repair(NER), and is also a part of the transcription factor II H(TFIIH) [8]. Study in benzene-exposed workers has shown that genetic variation in *ERCC3* may contribute to individual susceptibility to benzene-induced hematotoxicity [9]. It has been reported that DNA methylation plays an important role in the regulation of gene expression [10,11,12]. DNA methylation at specific CpG sites may alter the binding affinity of important transcription factors [10,13]. We previously found that average methylation level of *ERCC3* promoter was increased in benzene-exposed workers compared to unexposed controls [14]. To test whether specific CpG sites of *ERCC3* play an important role in benzene-induced epigenetic changes, and whether the specific methylation patterns are associated with benzene hematotoxicity, we analyzed the CpG methylation levels in *ERCC3* promoter region and transcription factor binding motif as well as the correlation between aberrant methylation and hematotoxicity.

## 2. Materials and Methods

### 2.1. Study Population and Biological Sample Collection

The study population is the same as that in our previous report [14], which included 76 workers exposed to benzene and 24 age- and sex-matched unexposed controls recruited from Tianjin and Shanghai, China. Briefly, benzene-exposed workers included 41 workers who engaged in painting, shoe making and printing, and had histories of benzene poisoning (BP) diagnosed by local Occupational Diseased Diagnostic Teams, and 35 healthy exposed workers without BP who worked in the same workplaces and had the same exposure duration (±5 years) as those with BP. The unexposed controls were selected from two workplaces: a clothing factory in Tianjin and an electric fan plant in Shanghai, China. The study was approved by the Ethical Review Committee in the National Institute for Occupational Health and Poison Control, Chinese Center for Disease Control and Prevention (China CDC). Participation was voluntary, and signed informed consent forms were obtained. Cumulative exposures were calculated by summing the workplace estimates over the exposure duration. Peripheral bloods were collected and analyzed for complete blood counts and differentials.

### 2.2. DNA Methylation Analysis

The DNA methylation analysis was described in detail by Xing et al. [14]. In brief, DNA methylation at CpG sites was quantified by the MassArray system (Sequenom EpiTYPER assay, San Diego, CA, USA) after isolating genomic DNA from peripheral blood. After the genomic DNA was treated with bisulfite, DNA amplification with T7-promoter tagged primers was preformed; PCR products were used to generatein vitro transcription and then subjected to base-specific cleavage with RNase A. All cleavage products were analyzed by matrix-assisted laser desorption/ionization time-of-flight mass spectrometry (MALDI-TOF MS) according to the manufacturer’s instructions. Then, the Sequenom EpiTYPER software converted the mass signals of the cleavage products to quantitative percent of methylated CpG sites. There were one CpG or more than one CpG contained in a cleavage product due to small DNA fragments.The cleavage products harboring one or more CpG sites were called CpG units. Human HCT116 DKO methylated and non-methylated DNA (Zymo Research, Irvine, CA, USA) were used as built-in positive and negative controls, respectively, to verify the efficiency of bisulfite-mediated conversion of DNA. Sixteen effective CpGs sites (10 CpG units) for the *ERCC3* were analyzed by Sequenom EpiTYPER (Sequenom, San Diego, CA, USA). We defined the Cm% as methylated cytosine percentage.

### 2.3. Target Prediction of Transcription Factors

To investigate the potential effect of methylation at CpG sites in promoter regions on gene transcriptional regulation, we analyzed transcription factor binding sites (TFBSs) and histone modification marks for the 16 CpG sites in the *ERCC3* promoter region based on human Refseq annotation. Experimentally validated transcription factors and histone modifications by ChIP-seq were obtained from Encyclopedia of DNA Elements (ENCODE) annotation in genome browser of University of California Santa Cruz (UCSC) [15]. Predictive transcription factors were obtained from JASPAR CORE Database (http://jaspar.genereg.net) (with 83% threshold of relative profile score) [16].

### 2.4. Statistical Analysis

As in our previous report [14], the methylation measures were logit-transformed and the WBC counts and percentage of neutrophil were log10-transformed to obtain an approximate normal distribution. We used linear regression models to examine the difference in DNA promoter methylation of *ERCC3* between benzene exposure and unexposed control with adjustment for age, sex, smoking status, alcohol drinking, body mass index (BMI), and percentage of lymphocytes, neutrophils and monocytes. Linear regression was also used to assess the association between DNA methylation levels of specific CpG sites and blood cell counts with adjustment of the same above covariates and other DNA methylation levels of CpG sites at the same promoter region. A two-tailed *p*-value less than 0.05 was considered significant. False discovery rate (FDR) was reported to account for multiple testing [17]. Data were analyzed using SPSS 11.5 software (IBM, Chicago, IL, USA).

## 3. Results

### 3.1. Demographic Characteristics and Benzene Exposure of the Study Population

As we previously reported [14], participants were matched for age, sex, smoking status and alcohol drinking, and therefore did not differ in these characteristics. The mean exposure duration (±SD) for the 76 exposed workers was 20 ± 9 years. The cumulative exposure levels for the exposed workers were more than 100 ppm-years based on the monitoring data from workplaces and estimation by trained field personnel, in which the mean exposure duration (±SD) for the BP patients was 19 ± 9 years. The mean interval of time (±SD) for the BP patients from last exposure to sample collection was 21 ± 10 years.

### 3.2. Methylation of Gene ERCC3

**Figure 1:**
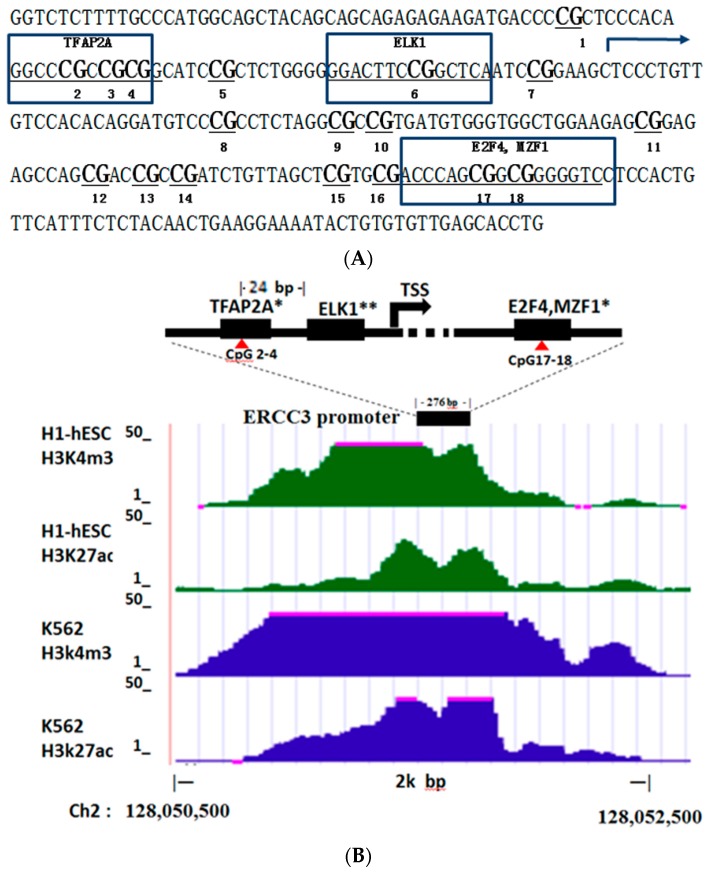
(**A**) The CpG dinucleotides contained TFBSs in the investigated sequence for methylation of *ERCC3* promoterregion. (**B**) The TFBSs and enrichment of H3K4me3 and H3K27ac modification in *ERCC3* promoter region in human embryonic stem cells (hESC) (Green area) and in K562 cells (Blue area). Red triangle indicated CpG unit. * TFBSs by JASPAR CORE Database (http://jaspar.genereg.net/). ** HMR Conserved TFBSs by Transfac Matrix Database (v.7.0) (https://genome.ucsc.edu/). TFAP2A: transcription factors activating enhancer binding protein 2 alpha, MZF1: myeloid zinc finger-1, TSS: transcriptional start site, TFBSs: transcription factor binding sites.

DNA methylation levels were analyzed for 16 CpG sites (10 CpG units), covering 276 bp of the human *ERCC3* promoter region. All the 10 CpG units had higher methylation levels in the workers exposed to benzene, two of which, located 43 bp upstream (CpG 2–4) and 99 bp downstream (CpG 17–18) of the transcription start site (TSS), showed significantly increased methylation levels in benzene-exposed workers compared with unexposed controls (Mean ± SD: 5.86 ± 2.77% vs. 4.92 ± 1.53%, *p* = 0.032; 8.45 ± 4.09% vs. 6.79 ± 2.50%, *p* = 0.024, respectively, Table 1). Further investigation of the two CpG units using the JASPAR CORE revealed that CpG2–4 contained putative binding sites for the transcription factors activating enhancer binding protein 2 alpha (TFAP2A), CpG 17–18 contained putative binding sites for the transcription factors (E2F4) and myeloid zinc finger 1 (MZF1) (Figure 1A,B). We also noted that an experimentally validated transcription factor ELK1 was binding to the CpG 6 site, which was located 19 bp upstream of TSS and 24 bp downsteam of CpG 2–4, while using ENCODE annotation from the UCSC genome browser. The methylation level of CpG 6 site was higher in workers exposed to benzene than unexposed controls although the difference was not significant (Table 1, Figure 1A,B). Since histone modifications in the promoter region were associated with transcription activity, we further investigated the tested 276-bp regions using the ENCODE annotation. The results showed that histone H3 lysine 4 trimethylation (H3K4me3) and histone H3 lysine 27 acetylation (H3K27ac) were significantly enriched in this region in K562 cells and human embryonic stem cells [18] (Figure 1B). The increased methylation levels of these specific CpG sites may inhibit *ERCC3* transcription by blocking transcription factors binding and effecting histone modifications.

### 3.3. Correlation between Hematotoxicity and Aberrant CpG Methylation Induced by Benzene

Hematotoxicity in workers exposed to benzene have been detailed previously [14]. Briefly, WBC counts were significantly decreased in benzene-exposed group compared with unexposed group (Mean ± SD: 5.0 ± 1.4 × 10^9^/L vs. 5.9 ± 1.4 × 10^9^/L, *p* = 0.01). Both absolute and relative numbers of neutrophils were decreased in exposed workers compared with unexposed controls (Mean ± SD: 58.1 ± 13.8% vs. 62.3 ± 8.2%, *p* = 0.08; 3.0 ± 1.0 × 10^9^/L vs. 3.6 ± 1.1 × 10^9^/L, *p* = 0.03, respectively), and the other absolute and relative numbers of lymphocytes, monocytes, eosinophils, and basophils were similar between exposed and unexposed subjects.

As we reported previously, methylation of specific CpG sites negatively correlated with tumor suppressor genes p15 mRNA expression, which positively correlated with hematotoxicity caused by benzene [5,19]. Next, we analyzed the correlations between the CpG methylation and hematotoxicity. We found that there was significantly negative correlation between the methylation levels of CpG 2–4 and the percentage of neutrophils (*β* = −0.676, *R*^2^ = 0.913, *p* = 0.005) after adjustment for sex, age, alcohol drinking, smoking status, body mass index, exposure duration, the methylation of the other 9 CpG units and the percentage of lymphocytes and monocytes in benzene-exposed workers. No other CpG sites in the *ERCC3* promoter region correlated with WBC counts or the percentage of lymphocytes, neutrophils and monocytes in exposed workers and controls.

## 4. Discussion

In a previous study, we reported that increased average methylation level of *ERCC3* was associated with benzene exposure [14]. Among the 76 benzene-exposed workers, 41 workers had a prior history of BP and 35 workers had no BP history. There were no significant differences between the two groups for the methylation levels of *ERCC3* (Mean ± SD: 4.39 ± 3.42% vs. 5.11 ± 3.51%, *p* = 0.608) after adjusting for potential confounders. In this study, we further investigated the methylation levels of the 16 CpG sites in the *ERCC3* promoter region for the same study subjects. We found that two CpG units (CpG 2–4 and 17–18), located 43 bp upstream and 99 bp downstream of the transcription start site (TSS) of *ERCC3*, respectively, had higher methylation levels in benzene-exposed workers than in unexposed controls. The higher methylation levels of CpG 2–4 in the *ERCC3* promoter region showed significantly negative correlation with the percentage of neutrophils in benzene-exposed workers.

Studies reported that hypermethylation of specific CpG sites in promoter region results in reduction of gene expression. Analysis of human renal cell carcinoma samples showed that the increased methylation levels of two specific CpG sites in Tensin3 gene promoter were correlated with lower Tensin3 gene expression [13]; Occupational exposure to polycyclic aromatic hydrocarbons induced hypermethylation of 22 specific CpG sites of *p16^INK4α^* and the correlation between hypermethylation and suppression of *p*16 were found in vitro [12]. Moreover, it has been suggested that DNA methylation near the TSS has a major impact on gene activity [20]. The methylation of the CpG site located at position −182 bp relative to TSS in the insulin gene *Ins2* promoter independently suppressed *Ins2* promoter activity by 50% [21], and the in vitro methylation of the promoter constructed at −111, −181 and −210 bp could completely inhibit the activity of Podocalyxin (Podx1) promoter [22]. In the present study, the CpG 2–4 and CpG17–18 were all located within 100 bp of the TSS. The CpG 2–4 and CpG 17–18 co-localise with 3 putative transcription factors, which are involved in cell proliferation and differentiation (TFAP2A) [23], cell cycle (E2F4) [24] and hematopoiesis (MZF1) [25]. We also found that an experimentally validated transcription factor ELK1 was bound to CpG 6, which was located 19 bp upstream of TSS and 24 bp downstream of CpG 2–4, and had higher methylation in benzene-exposed workers than unexposed controls, although the difference was not significant. Since the methylated CpG sites has the potential to block transcription factor (TF) binding through interference with base recognition [26], the increased methylation levels of these CpG sites near the TSS may therefore play an important role in inhibiting *ERCC3* transcription in benzene-exposed workers, thus contribute to genomic instability. Our previous study reported a significant negative correlation between the methylation of specific CpG sites and mRNA expression levels in the tumor suppressor genes *p15* and *p16* in benzene poisoning patients [5]. In this study, we performed the expression analysis in human acute promyelocytic leukemia cells (HL60) in the presence of hydroquinone (HQ), a key benzene toxic metabolite, and found that HQ can induce down-regulation of *ERCC3* after 72 h treatment (data not shown). Our findings provide a potential molecular mechanism for the observed association between increased promoter methylation and decreased mRNA expression of *ERCC3*. Additional studies involving the methylation and expression analysis in workers exposed to benzene are needed to confirm these findings.

A recent study found specific methylation patterns in CpG islands in different celltypes during selective events. The effect of methylation on chromatin structure may contribute to transcriptional regulation [27]. Using the ENCODE annotation from the UCSC genome browser, we found that histone H3K4me3 and H3K27ac were significantly enriched in the tested 276-bp region within *ERCC3* promoter in both K562 cells and human embryonic stem cells [18]. Our results suggest that the individual methylation events for each CpG site and the chromatin modification along with methylation worth to be investigated in vitro/vivo to confirm the effect of benzene on methylation and gene transcription of *ERCC3*.

As a key player in NER, *ERCC3* is responsible for repairing bulky DNA adducts formed by benzene. *ERCC3* mutation is associated with xeroderma pigmentosum [28] and breast cancer [29]. A study in benzene-exposed workers reported that single nucleotide polymorphisms (SNP) in the *ERCC3* gene region were both associated with altered WBC and granulocyte counts [9]. Total WBC <4000/µL or neutrophil count <2000/µL are one of the key factors in the diagnostic criteria for occupational benzene poisoning according to the Ministry of Health of the People’s Republic of China [30]. Our results showed that the WBC counts and the percentage of neutrophils were lower in benzene-exposure workers compared with the unexposed controls; however, we did not find any correlation between WBC counts and DNA methylation of *ERCC3*. Interestingly, we found that increased DNA methylation levels of CpG 2–4 in the *ERCC3* promoter region were associated with a decreased percentage of neutrophils in benzene-exposed workers. The present result supplements the findings of our previous studies, in which we demonstrated that the *p15* mRNA expression negatively correlated with increased methylation at specific CpG sites [5] and positively correlated with WBC counts and neutrophil counts [19]. Further genome-wide methylation studies with larger number of samples will be required to assess the role of DNA methylation in benzene induced hematotoxicity.

Given that granulocytes, lymphocytes and monocytes have unique DNA methylation signature, which may act as a potential confounding factor in investigation of DNA methylation [31], we adjusted cell proportion using linear regression models in methylation and correlation analysis; however, other lymphocyte subsets, such as T cell, B cell and NK cell, which may be involved in the aberrant methylation and the correlation, cannot be ruled out. A lack of correlation between WBC counts and DNA methylation could reflect the small number of subjects in this study. In addition, it has been shown that DNA methylation can be modified by folate, a key mediator in the transfer of one carbon group for DNA methylation [32]. Lower folate levels in diet caused dysregulation of DNA methylation and played an important role in vascular disease and tumorigenesis [33,34]. Several studies have also shown that methylenetetrahydrofolate reductase (MTHFR) polymorphism is associated with aberrant genomic DNA methylation in human with lower folate levels [35,36]. MTHFR C677T polymorphism interacted with folate to influence CpG promoter methylation [37]. Unfortunately we lacked precise individual benzene exposure data limiting our investigation of dose-response associations. In future studies, the accurate exposure estimation is needed to minimize the individual variation and measurement bias. Taken together, additional studies taking into consideration these factors are necessary to reach a more definite conclusion.

## 5. Conclusions

In conclusion, DNA methylation of the two specific CpG sites in the *ERCC3* promoter region were increased in the workers exposed to benzene compared to unexposed controls. Moreover, the increased methylation levels of specific CpG sites in the *ERCC3* promoter region were associated with decreased percentage of neutrophils in benzene-exposed workers. Our study suggests that the methylation of specific CpG sites of *ERCC3* may serve as a potential epigenetic marker for risk assessment of occupational exposure to benzene.

## Figures and Tables

**Table 1:** Associations between benzene exposure and promoter DNA methylation in *ERCC3*.

CpG Unit ^a^	In Relation to TSS	Control (*n* = 24) Mean ± SD (Cm%)	Exposure (*n* = 76) Mean ± SD (Cm%)	Beta	SE	*p* Value	FDR Value
1	−62 bp	3.04 ± 2.69	3.87 ± 3.94	0.07	0.19	0.725	0.725
2–4	−43 bp	4.92 ± 1.53	5.86 ± 2.77	0.19	0.09	0.032 *	0.176
5	−36 bp	1.38 ± 1.66	2.51 ± 3.44	0.32	0.18	0.082	0.226
6	−19 bp	1.21 ± 2.02	2.74 ± 3.90	0.28	0.20	0.153	0.281
7	−9 bp	4.54 ± 3.11	4.69 ± 4.03	0.09	0.18	0.626	0.689
9–10	+30 bp	6.96 ± 3.13	7.74 ± 3.49	0.15	0.09	0.107	0.235
11	+56 bp	1.15 ± 1.68	2.81 ± 4.36	0.21	0.38	0.576	0.689
13–14	+71 bp	6.46 ± 4.96	7.47 ± 5.50	0.19	0.19	0.317	0.436
15–16	+87 bp	1.54 ± 2.06	3.04 ± 3.85	0.39	0.20	0.059	0.216
17–18	+99 bp	6.79 ± 2.50	8.45 ± 4.09	0.24	0.11	0.024 *	0.176

^a^ data missing due to low signal/noise ratio by MALDI-TOF MS. Cm%: methylated cytosine percentage; FDR: false discovery rate. Linear regression models were adjusted for sex, age, alcohol drinking,smoking status, body mass index, and percentage of lymphocytes, neutrophils and monocytes. * *p* value < 0.05.
